# Use of a Data Repository to Identify Delirium as a Presenting Symptom of COVID-19 Infection in Hospitalized Adults: Cross-Sectional Cohort Pilot Study

**DOI:** 10.2196/43185

**Published:** 2023-11-30

**Authors:** Laurence M Solberg, Laurie J Duckworth, Elizabeth M Dunn, Theresa Dickinson, Tanja Magoc, Urszula A Snigurska, Sarah E Ser, Brian Celso, Meghan Bailey, Courtney Bowen, Nila Radhakrishnan, Chirag R Patel, Robert Lucero, Ragnhildur I Bjarnadottir

**Affiliations:** 1 Geriatrics Research, Education, and Clinical Center North Florida/South Georgia Veterans Health System Veterans Health Administration Gainesville, FL United States; 2 College of Nursing University of Florida Gainesville, FL United States; 3 Shands Hospital UF Health Gainesville, FL United States; 4 Department of Epidemiology College of Public Health and Health Professions University of Florida Gainesville, FL United States; 5 College of Medicine University of Florida Gainesville, FL United States; 6 College of Medicine University of Florida Jacksonville, FL United States; 7 School of Nursing University of California Los Angeles Los Angeles, CA United States

**Keywords:** COVID-19, delirium, neurocognitive disorder, data repository, adults, pilot study, symptom, electronic health record, viral infection, clinical, patient, research, diagnosis, disorder, memory, covid, memory loss, old, old age

## Abstract

**Background:**

Delirium, an acute confusional state highlighted by inattention, has been reported to occur in 10% to 50% of patients with COVID-19. People hospitalized with COVID-19 have been noted to present with or develop delirium and neurocognitive disorders. Caring for patients with delirium is associated with more burden for nurses, clinicians, and caregivers. Using information in electronic health record data to recognize delirium and possibly COVID-19 could lead to earlier treatment of the underlying viral infection and improve outcomes in clinical and health care systems cost per patient. Clinical data repositories can further support rapid discovery through cohort identification tools, such as the Informatics for Integrating Biology and the Bedside tool.

**Objective:**

The specific aim of this research was to investigate delirium in hospitalized older adults as a possible presenting symptom in COVID-19 using a data repository to identify neurocognitive disorders with a novel group of *International Classification of Diseases, Tenth Revision* (ICD-10) codes.

**Methods:**

We analyzed data from 2 catchment areas with different demographics. The first catchment area (7 counties in the North-Central Florida) is predominantly rural while the second (1 county in North Florida) is predominantly urban. The Integrating Biology and the Bedside data repository was queried for patients with COVID-19 admitted to inpatient units via the emergency department (ED) within the health center from April 1, 2020, and April 1, 2022. Patients with COVID-19 were identified by having a positive COVID-19 laboratory test or a diagnosis code of U07.1. We identified neurocognitive disorders as delirium or encephalopathy, using ICD-10 codes.

**Results:**

Less than one-third (1437/4828, 29.8%) of patients with COVID-19 were diagnosed with a co-occurring neurocognitive disorder. A neurocognitive disorder was present on admission for 15.8% (762/4828) of all patients with COVID-19 admitted through the ED. Among patients with both COVID-19 and a neurocognitive disorder, 56.9% (817/1437) were aged ≥65 years, a significantly higher proportion than those with no neurocognitive disorder (*P*<.001). The proportion of patients aged <65 years was significantly higher among patients diagnosed with encephalopathy only than patients diagnosed with delirium only and both delirium and encephalopathy (*P*<.001). Most (1272/4828, 26.3%) patients with COVID-19 admitted through the ED during our study period were admitted during the Delta variant peak.

**Conclusions:**

The data collected demonstrated that an increased number of older patients with neurocognitive disorder present on admission were infected with COVID-19. Knowing that delirium increases the staffing, nursing care needs, hospital resources used, and the length of stay as previously noted, identifying delirium early may benefit hospital administration when planning for newly anticipated COVID-19 surges. A robust and accessible data repository, such as the one used in this study, can provide invaluable support to clinicians and clinical administrators in such resource reallocation and clinical decision-making.

## Introduction

Delirium, an acute confusional state highlighted by inattention [1,2], has been reported to occur in 10% to 50% of patients with COVID-19 [3,4]. People hospitalized with COVID-19 have been noted to present with or develop delirium and neurocognitive disorders [5,6]. Delirium has been noted as a common presentation to emergency departments (EDs) during the current pandemic but is an atypical symptom of COVID-19 in hospitalized patients [6]. Recognizing delirium as a possible presenting symptom of COVID-19 may decrease the effects on the patient and the health care system.

Delirium has been found to be the most common neurocognitive syndrome in the acute hospital setting and is a condition of particular concern due to its association with multiple adverse outcomes, including increased critical care use and costs, prolonged length of stay, increased rate of discharge to skilled nursing facilities, long-term cognitive decline, greater functional impairment, increased numbers of readmission, and increased mortality [7-12].

Caring for patients with delirium is associated with more burden for nurses, clinicians, and caregivers [13,14] and poorer clinical outcomes for patients [15]. Fortunately, delirium has been able to be managed through multicomponent interventions including nonpharmacologic ones [16-18]; however, the cost and personnel required for these multicomponent prevention programs can be prohibitive and requires a way of identifying patients at the greatest risk for developing delirium [19].

The COVID-19 pandemic has increased demands on nurses and other hospital clinical resources and intensified the possible consequences of delirium in hospitalized people. Additional infection control precautions, isolations, and personal protective equipment conservation efforts have only amplified the difficulty of delivering multicomponent interventions that have been shown to reduce delirium risk [20,21]. This combination of circumstances, which developed during care of the various surges of patients with COVID-19, increased the urgency to develop alternate means of determining delirium risk through new advances in technologies and data science.

Using information in electronic health record (EHR) data to recognize delirium and possibly COVID-19 could lead to earlier treatment of the underlying viral infection and improve outcomes in clinical and health care systems cost per patient. However, clinical EHR data are largely inaccessible due to data sensitivity, ethical issues, and data complexity [22], hindering the rapid discovery necessary to recognize health trends and generate knowledge during emergent situations such as a pandemic. Clinical data repositories or data warehouses address part of this issue by integrating and organizing information from EHR systems. While EHR systems are mainly optimized for clinical transaction processing and intramural systems communication, a clinical data repository allows information to be organized in a database form that can be tailored for retrieval and analysis. Clinical data repositories can further support rapid discovery through cohort identification tools, such as the Informatics for Integrating Biology and the Bedside (i2b2) tool. The National Institute of Health funded the i2b2 National Center for Biomedical Computing to provide an open-source framework for informatics tools that facilitate clinical data analysis and integration [22,23]. The tool can support self-service and custom queries on deidentified data, protecting patient privacy without requiring a lengthy scientific review process for each query. Information accessible through i2b2 has been reported to provide more realistic cohort identification for clinical trials, expose a new data source for hypothesis generation, and provide a foundation for vendor neutral interinstitutional research [23,24]. Less attention has been devoted to the potential utility of cohort identification tools such as i2b2 for clinicians and hospital systems to identify trends, generate knowledge to improve care, or inform resource allocation.

It has been noted that delirium is often underdiagnosed and undercoded. However, EHR data have shown to be valid to study occurrence of delirium [25-28]. Documentation and data from the EHR during routine care have been used and validated in developing clinical prediction models of delirium risk prior to and during this pandemic [28-31]. Using cohort identification tools for rapid detection and exploration of trends could enable the mitigation of pandemic-related stressors on health systems and improve treatments and outcomes. The specific aim of this research was to investigate delirium in hospitalized older adults as a possible presenting symptom in COVID-19 using a data repository (i2b2) to identify neurocognitive disorders with a novel group of *International Classification of Diseases, Tenth Revision* (ICD-10) codes.

## Methods

### Ethics Approval

The University of Florida’s institutional review board approved this cross-sectional exploratory retrospective study using secondary data analysis (IRB-201900208).

### Data Collection

Our data were extracted from a HIPAA (Health Insurance Portability and Accountability Act) limited repository of EHR data, i2b2 [22], and supplemented by counts provided by an honest broker team at a large comprehensive academic health center serving diverse communities across North and Central Florida. The health system serves more than 2 million patients from all 67 counties in Florida.

In this study, we analyzed data from 2 catchment areas with different demographics. The first catchment area (7 counties in North-Central Florida) is predominantly rural and consists of approximately 906,697 people (n=144,867, 16% Black; n=679,582, 75% White; and n=82,248, 9.1% other, including n=26,043, 2.9% Asian). The Hispanic or Latino population in this catchment area is estimated to be 108,462 (12%). The second catchment area (1 county in North Florida) is predominantly urban and consists of approximately 857,191 people (n=276,791, 32.3% Black; n=498,033, 58.1% White; and n=82,367, 9.6% other, including n=47,888, 5.6% Asian). The Hispanic or Latino population in this catchment area is estimated to be 100,564 (11.7%).

The i2b2 data repository was queried for patients with COVID-19 admitted to inpatient units via the ED within the health center during the period between April 1, 2020, and April 1, 2022. Patients with COVID-19 were identified by having a positive result of a COVID-19 laboratory test or having a diagnosis code of U07.1. Further, the data repository was queried for a subset of patients with COVID-19 diagnosed with neurocognitive disorders during their hospitalizations. We identified neurocognitive disorders as delirium or encephalopathy, using the following ICD-10 codes: R41.0, R41.82, R41.9, F05, and F44.89 for delirium and G92, G92.8, G92.9, G93.40, G93.41, and G93.49 for encephalopathy. We further distinguished between diagnostic codes present on admission (PoA) that were defined as patients’ condition on admission to the hospital; discharge diagnosis that was defined by hospital coder; and any diagnosis that included discharge diagnosis, physician billing diagnosis, and problem list diagnosis. All queries included demographics (ie, age, gender, race, and ethnicity).

Due to limited data availability in the i2b2, data were augmented by counts provided by an honest broker team at our institution. While the i2b2 data repository contains only a subset of data elements from the EHR, the honest broker team has access to the entire EHR system. The additional data included average length of stay and mortality rate for each cohort that we considered in this study.

We used the obtained data to produce descriptive statistics, including frequencies and percentages, and examined the data by individual diagnoses, as well as within time intervals corresponding to the national peaks of the Alpha (March 27, 2021, to June 12, 2021), Delta (July 3, 2021, to September, 30, 2021), and Omicron (December 1, 2021, to April 1, 2022) variants. We defined peak as an interval when at least 50% of national cases belonged to a specific variant. We used the i2b2 tool for counts of unique patients satisfying the inclusion diagnoses. This approach resulted in each patient being counted only once within a specific time interval even if the patient met the inclusion criteria during multiple hospital encounters. Bivariate associations were analyzed using an “N-1” chi-square test [32,33] or a 2-tailed Welch *t* test (used for length of stay) [34]. Bivariate associations were considered statistically significant with a *P* value of .05 or less. Statistical tests were performed using *R* (version 4.2.0; *R* Foundation for Statistical Computing).

## Results

A total of 4828 patients were hospitalized with COVID-19 through the ED during this study’s period. Table 1 displays the descriptive characteristics of these patients. Adults aged 65 years and older constituted 39.1% (n=1886) of the COVID-19 hospitalizations, and 50.7% (n=2449) of patients hospitalized with COVID-19 were male. Black patients represented 29.7% (n=1432) of all COVID-19 hospitalizations; additionally, 6.6% (n=318) of patients were Hispanic or Latino. The average length of stay was 9.6 days. The admission ended in death for 10.5% (n=507) of patients hospitalized with COVID-19 through the ED during this study’s period.

Slightly less than one-third (n=1437, 29.8%) of patients with COVID-19 were diagnosed with a co-occurring neurocognitive disorder, that is, delirium or encephalopathy. Among patients with both COVID-19 and a neurocognitive disorder, 56.9% (817/1437) were aged 65 years or older, a significantly higher proportion than those with no neurocognitive disorder (*P*<.001). There was also a significantly higher proportion of male patients in this group, compared to those with no neurocognitive disorder (*P*=.02). Just under a third (418/1437, 29.1%) of patients with both COVID-19 and a neurocognitive disorder expired during their hospital stay, which is a significantly higher proportion than among those with COVID-19 and no neurocognitive disorder (*P*<.001). The length of stay in this group was also substantially higher (20.8 days).

**Table 1:** Patient characteristics.

Characteristics		All patients with COVID-19 (N=4828), n (%)	Patients with COVID-19 and neurocognitive disorder (n=1437), n (%)	Patients with COVID-19 but no neurocognitive disorder (n=3391), n (%)	*P* value
**Age group^a^ (years)**					
	<65	2942 (60.9)	620 (43.1)	2322 (68.5)	<.001
	≥65	1886 (39.1)	817 (56.9)	1069 (31.5)	<.001
**Sex^a^**					
	Female	2379 (49.3)	669 (46.6)	1710 (50.4)	.02
	Male	2449 (50.7)	768 (53.4)	1681 (49.6)	.02
**Race**					
	Black	1432 (29.7)	409 (28.5)	1023 (30.2)	.24
	White	2981 (61.7)	916 (63.7)	2065 (60.9)	.07
	Other	415 (8.6)	112 (7.8)	303 (8.9)	.21
**Ethnicity**					
	Hispanic	318 (6.6)	80 (5.6)	238 (7)	.07
	Non-Hispanic	4437 (91.9)	1322 (92)	3115 (91.9)	.91
	Other	73 (1.5)	35 (2.4)	38 (2.2)	.67
Expired^a^		507 (10.5)	418 (29.1)	89 (5.3)	<.001
Length of stay (days)^a^, mean (SD)		9.6 (13.5)	20.8 (24.3)	4.9 (6)	<.001

^a^Indicates that the variable was statistically significant at the .05 significance level.

A neurocognitive disorder was PoA for 15.8% (762/4828) of all patients with COVID-19 admitted through the ED. Table 2 shows a comparison of sample characteristics for patients with COVID-19 where the co-occurring neurocognitive disorder was PoA versus diagnosed later during the hospitalization. Among the 1437 patients with both COVID-19 and a neurocognitive disorder, just over half (762/1437, 53%; *P*<.001) had the neurocognitive disorder PoA. Significantly higher proportion of patients with COVID-19 and a neurocognitive disorder PoA were aged 65 years or older (485/762, 64%; *P*<.001) and Black (238/762, 31.2%; *P*=.01), compared to those whose neurocognitive disorder was not assigned as PoA (171/675, 25.3%). Contrastingly, significantly lower proportions of patients with COVID-19 and a neurocognitive disorder PoA identified as other race (49/762, 6.4% vs 63/675, 9.3%; *P*=.04) and Hispanic (33/762, 4.3% vs 47/675, 7%; *P*=.03). A significantly higher proportion of patients whose neurocognitive disorder was not assigned as PoA expired during their hospitalization (*P*<.001).

**Table 2:** Comparison of patient characteristics between those with neurocognitive disorder PoA^a^ versus those diagnosed later during the hospitalization.

Characteristics		Patients with COVID-19 and a neurocognitive disorder PoA (n=762), n (%)	Patients with COVID-19 and a neurocognitive disorder not PoA (n=675), n (%)	*P* value
**Age group^b^ (years)**				
	<65	277 (36.4)	343 (50.8)	<.001
	≥65	485 (63.6)	332 (49.2)	<.001
**Sex**				
	Female	361 (47.4)	308 (45.6)	.49
	Male	401 (52.6)	367 (54.4)	.49
**Race**				
	Black^b^	238 (31.2)	171 (25.3)	.01
	White	475 (62.3)	441 (65.3)	.24
	Other^b^	49 (6.4)	63 (9.3)	.04
**Ethnicity**				
	Hispanic^b^	33 (4.3)	47 (7)	.03
	Non-Hispanic	709 (93)	613 (90.8)	.13
	Other	20 (2.6)	15 (2.2)	.62
Expired^b^		177 (23.2)	241 (35.7)	<.001
Length of stay (days)^b^, mean (SD)		15.1 (19.3)	27.2 (26.2)	<.001

^a^PoA: present on admission.

^b^Indicates that the variable was statistically significant at the .05 significance level.

Among the 1437 patients with COVID-19 and a neurocognitive disorder, 290 (20.2%) of them were diagnosed with delirium only, 470 (32.7%) were diagnosed with encephalopathy only, and 677 (47.1%) were diagnosed with more than 1 neurocognitive disorder. The proportion of patients younger than 65 years (239/470, 50.9%; *P*<.001) was significantly higher among patients diagnosed with encephalopathy only compared with patients diagnosed with delirium only (103/290, 35.5%; *P*<.001) and both delirium and encephalopathy (278/677, 41.1%; *P*<.001; Multimedia Appendix 1).

Significant differences were observed in the proportion of patients who expired during their hospitalization. Among the 418 patients with COVID-19 and neurocognitive disorders who expired during their hospitalization, half (209/470, 44.5%) of them were diagnosed with only encephalopathy. For comparison, 28.2% (191/677) of those diagnosed with more than 1 neurocognitive disorder and 6.2% (18/290) of those diagnosed with delirium alone expired during their hospitalization. In terms of length of stay, patients diagnosed with more than 1 neurocognitive disorder had the longest average length of stay at 23.4 days, followed by patients diagnosed with encephalopathy alone at 18.9 days. The average length of stay for patients diagnosed with delirium alone was 12.1 days.

Most (1272/4828; 26.3%) patients with COVID-19 admitted through the ED during our study period were admitted during the Delta variant peak. Among the 1437 patients with COVID-19 and neurocognitive disorder, 71 (4.9%) were admitted during the Alpha variant peak, 403 (28%) were admitted during the Delta variant peak, and 317 (22.1%) were admitted during the Omicron variant peak.

**Figure 1:**
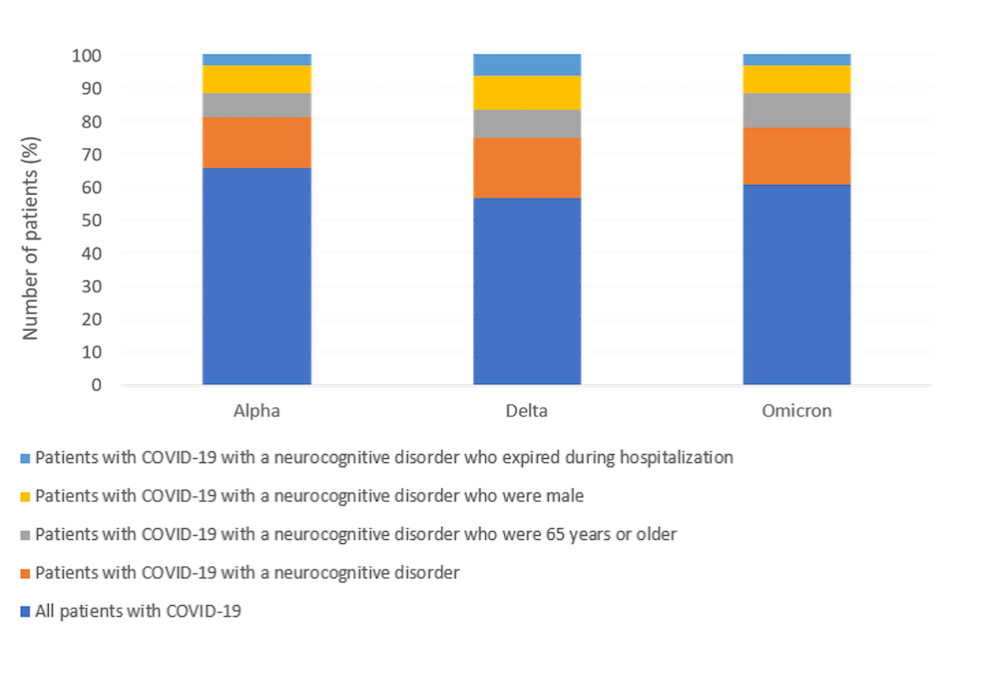
Counts across the 3 variant peaks for patients admitted through the emergency department who had COVID-19, COVID-19 and a neurocognitive disorder, COVID-19 and a neurocognitive disorder who were aged 65 years or older, COVID-19 and a neurocognitive disorder who were male, and COVID-19 and a neurocognitive disorder who expired during their admission.

A significantly higher proportion of those admitted during the Omicron variant peak were aged 65 years or older (191/317, 60.3%), compared to both Alpha and Delta variants (33/71, 47%; *P*=.03 and 191/403, 47.4%; *P*<.001, respectively). A significantly higher proportion of patients admitted during the Delta variant peak were male (229/403, 56.8%), compared to the Omicron variant peak (154/317, 48.6%; *P*=.03). In addition, a significantly higher proportion of patients admitted during the Delta variant peak expired during their hospitalization (147/403, 36.5%), compared to both Alpha and Omicron (16/71, 23%; *P*=.02 and 64/317, 20.2%; *P*<.001, respectively). Finally, the average length of stay was the shortest (15.8 days) among patients admitted during the Omicron variant peak (Figure 1).

## Discussion

### Principal Findings

The data collected demonstrated that an increased number of older patients, 65 years and older, with neurocognitive disorder PoA were infected with COVID-19.The presence of delirium or neurocognitive disorder was shown to be more than twice as high in this group than what normally is found in emergency departments and acute care settings (485/1437, 33.7%; *P*<.001) compared to the incidence rate of 9% to 14% in emergency departments that has been reported in the literature by Kennedy et al [35]. Furthermore, the average length of stay in patients with COVID-19 and delirium was shown to be 4 times as long. Of note, a much higher and statistically significant number of people with neurocognitive disorder and COVID-19 died than those with only COVID-19 (*P*<.001).

The data repository review showed that just over a third (103/290, 35.5%) of the cases of delirium and COVID-19 were in people younger than 65 years. While a higher number of patients over 65 years old had delirium PoA (in the ED), there was a higher percentage of patients younger than 65 years who developed delirium while in the hospital. Unfortunately, not all the admitted patients were tested for COVID-19 on admission, particularly in the early part of the pandemic. Nevertheless, this suggests that it may be beneficial to adopt delirium prevention protocols for all patients with COVID-19 upon admission.

Social isolation among individuals aged 65 years and older has been associated with future ED use and poorer health outcomes [36]. However, Falcão et al [37] showed that the number of visits to ED per day decreased by 45% during lockdown and nonelective hospital admissions decreased by 50% during periods of social isolation. One possible explanation of the prevalence of delirium PoA, particularly among older adults, is the social isolation people experienced during the pandemic. This may have led to people waiting longer before presenting to the ED due to the fear of going to the hospital. The psychosocial implications of social isolation during a pandemic warrant further study.

Our data show that approximately 23.9% (421,658/1,763,888) of the patients in our catchment areas are Black, yet 29.7% (1432/4828) of the patients being admitted were Black. Our data also show a higher proportion of Black patients being admitted for COVID-19. These results coincide with past research that describes racial disparities in COVID-19 cases. Black and Hispanic patients were shown to be disproportionately affected by COVID-19 [38] in both hospitalizations and deaths rates. Moreover, this further validates the use of a data repository to investigate health care disparities.

A data repository to review incidence of delirium coinciding with COVID-19 may help hospitals align resources to COVID-19 units to decrease strain on an already stressed system with limited resources. Currently, the data repository we used refreshes i2b2-accessible data every 90 days, which can provide useful information for hospital or health systems for quarterly planning of staffing and resource reallocation. However, the fast-paced environment of modern health care requires more current data, particularly in a rapidly evolving public health emergency such as the COVID-19 pandemic. Future research is needed to explore and develop the technical infrastructure required to provide real-time or near real-time data to support resource allocation and clinical decision-making, as part of a learning health care system.

Furthermore, future studies should examine the role of any neurocognitive symptoms on presentation to the ED in diagnosing COVID-19. Further, 1 hypothesis to explore is whether neurocognitive symptoms on presentation to the ED should be considered COVID-19 unless ruled out by testing. Finally, length of stays related to delirium and allocation of resources can be investigated to discover more efficient use of hospital resources. With a robust data science infrastructure, innovative artificial intelligence solutions can be implemented to address these issues and improve care for patients having COVID-19 and delirium, as well as emerging future health care threats.

### Limitations

A limitation of this study is that not all the admitted patients were tested for COVID-19 on admission. Therefore, the entry to the ED as a variable to developing delirium was not available. Another limitation is the lack of documentation for the presence of a preexisting neurocognitive disorder or a baseline mental status of the patients. An additional limitation is that our research did not attempt to factor in any vaccine effect during the different variant surges due to the lack of data on vaccination status. Further, a limitation is that the cause of death may not be easily available through data extraction with i2b2 and caution must be exercised when interpreting such data. These limitations highlight the trade-offs inherent in the use of cohort identification tools, as the ability for quick and direct queries without additional scientific review necessitates that the data available through such tools be limited to protect data privacy and confidentiality.

### Conclusions

Identifying delirium as a presenting sign of COVID-19 may be beneficial to better develop care plans for patients and resource planning for hospitals. Knowing that delirium increases the staffing, nursing care needs, hospital resources used, and the length of stay as previously noted, identifying delirium early may benefit hospital administration when planning for newly anticipated COVID-19 surges. During COVID-19 surges, it is imperative to use hospital resources efficiently and effectively to better meet the demands of patients in need of a higher level of care. If the length of stays related to delirium can be decreased, there exists the potential to reallocate resources where they are the most needed. A robust and accessible data repository, such as the one used in this study, can provide invaluable support to clinicians and clinical administrators in such resource reallocation and clinical decision-making.

## Appendix

Multimedia Appendix 1 Comparison of patients with COVID-19 who have delirium only, encephalopathy only, and more than 1 neurocognitive disorder.

## Abbreviations

ED: emergency department
EHR: electronic health record
HIPAA: Health Insurance Portability and Accountability Act
i2b2: Integrating Biology and the Bedside
*ICD-10*: *International Classification of Diseases, Tenth Revision*
PoA: present on admission
