# Supplementary figures and images for: Use of a Data Repository to Identify Delirium as a Presenting Symptom of COVID-19 Infection in Hospitalized Adults: Cross-Sectional Cohort Pilot Study
Source: JMIR Aging. 2023 Nov 30;6:e43185. doi: 10.2196/43185 (PMC10722366; doi:10.2196/43185)

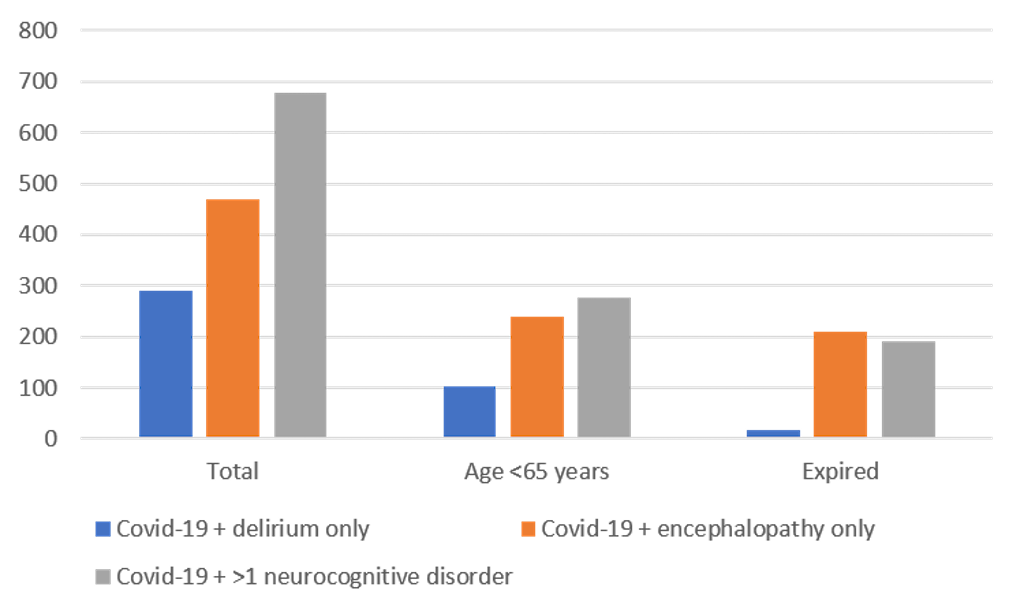

Supplement: Multimedia Appendix 1 [file aging_v6i1e43185_app1.png]
